# The suitable fixation for unstable intertrochanteric fractures: A protocol of comparative clinical study

**DOI:** 10.1097/MD.0000000000023046

**Published:** 2020-10-30

**Authors:** Yu Bo, Yue Qin, Yuan Zang, Haibo Yang

**Affiliations:** a Department of Trauma Orthopedics; b Department of Clinical Skills Training Center, General Hospital of Ningxia Medical University, Ningxia, China.

**Keywords:** unstable intertrochanteric fracture, proximal femoral nail antirotation, sliding hip screw, complication, protocol

## Abstract

**Background:**

Normally taking the surgery is the standard treatment, between sliding hip screw (SHS) and utilizing proximal femoral nail antirotation (PFNA) for intramedullary fixation, it is still not certain which one work better for this type of fracture. Our purpose for this study was to determine the optimum choice of implant for a patient with an unstable intertrochanteric fracture.

**Methods:**

In our hospital, a reflective analysis was conducted of all unstable intertrochanteric fractures treated with either a SHS or PFNA fixation between February 2015 and February 2018. The rules of choosing patients were the following: older age of more than 60, unstable intertrochanteric fractures, and willingness to take clinical and radiographic follow-up researches for over 12 months. In this case, patients bearing former hip surgery at any side were removed from the candidates. Demographic characteristics collected effective information including gender, date of operation, and other relevant information. Postoperative outcome measures included operation time, total blood loss amount, validated mobility score, surgery-related syndrome, and tip-apex length. The patients were re-examined at three time periods: 3 weeks, 3 months, and 1 year. The result of *P* < .05 was considered to be statistically significant.

**Results:**

We were able to directly compare the outcomes of PFNA vs SHS techniques and might reveal a better technique in treatment of an unstable intertrochanteric fracture.

**Trial registration:**

This study protocol has been registered in Research Registry (researchregistry6057).

## 1 Introduction

Intertrochanteric hip fracture is the most common kind of damage among the senior citizen, with an annual incidence of more than 150,000 reported in the United States.^[1]^ The occurring chance of femoral intertrochanteric fractures has been rising recently, and the resulting mortality rate stays at 30% within 5 years after the fracture.^[2–4]^ With the rise of life expectancy, the disease burden of intertrochanteric fractures also increases. Though equipment designed to treat such fractures, fixation failures have made much progress, excessive rehabilitation costs and the augmented time staying at hospital still exist.^[5,6]^ Normally taking the surgery is the standard treatment, between sliding hip screw (SHS) and utilizing proximal femoral nail antirotation (PFNA) for intramedullary fixation, it is still not certain which one work better for this type of fracture.^[7,8]^

A large number of prospective randomized trials have made it clear that the SHS device has the ability to heal most Intertrochanteric fracture, meanwhile the rate of implantation failure could be controlled to <5%.^[9–14]^ Parker and Handoll have conducted experiments to compare extramedullary devices with intramedullary devices in a meta-analysis consisting of more than 3500 patients and found out that there was no significant differences in death rate, possibility to get infection, incision, loss amount of blood, duration of operation, and radiotherapy time.^[15]^ Yu et al have been conducted a meta-analysis and show that When treating unstable intertrochanteric fractures in adults, the incidence of implantation and reoperation failure is lower, and postoperative functional recovery is better under the circumstance of using intramedullary fixation, and most postoperative complications are not statistically different from extramedullary fixation.^[16]^ However, many authors have suggested that SHS fixation is the best for stable fractures, PFNA may be the best for unstable fractures, and AO type A3 fractures should definitely be treated with intramedullary nails. These theories about fracture types and fixation methods have never been authenticated by clinical studies.^[17–19]^

The ideal fixation of unstable intertrochanteric fractures has been the topic of debate for a quite long time Restricted by the limited sample size, it was difficult to draw definite conclusions from previous studies. Our purpose for this study was to determine the optimum choice of implant for a patient with a unstable intertrochanteric fractures.

## 2 Materials and methods

### 2.1 Study design and population

In our hospital, a reflective analysis was conducted of all unstable intertrochanteric fractures treated with either a SHS or PFNA fixation between February 2015 and February 2018. The research was approved via the institutional review committee of General Hospital of Ningxia Medical University, with the number NX-10840. The research has also been registered in the Research Registry named researchregistry6057.

The rules of choosing patients were the following: older age of more than 60, unstable intertrochanteric fractures, and willingness to take clinical and radiographic follow-up researches for over 12 months. In this case, patients bearing former hip surgery at any side were removed from the candidates. Patients bearing multiple ipsilateral or contralateral fragmentation or pathological fractures, intracapsular fractures or stable fractures (AO type 1) and those unwilling to participate were excluded. Patients who were unable to walk, bedridden, or sitting in a wheelchair were also removed from the candidates.

### 2.2 Techniques

The operation was performed on the operation table adopting spinal anesthesia. During the operation of fracture reduction and implantation of the PFNA or SHS, the procedures were performed on a fracture table under image intensifier control. The technique was proximately the same as that depicted by Mereddy et al^[20]^ for PFNA and MacLean et al^[21]^ used for SHS. The PFNA nail utilized in this study is composed of solid titanium nails with a length ranging from 200 to 240 mm and a diameter of 9.0 to 10.0 mm. The SHS plate contained stainless steel and includes a 25 or 38 mm barrel and 3 to 12 holes. The length of the shaft is between 62 and 206 mm.

### 2.3 Postoperative care

Drugs able to reduce pain and inflammation, such as warfarin and aspirin were used for postoperative thrombosis prevention. Same standardized postoperative multimodal pain regimen was given to all the patients, which contained 1 g acetaminophen, 200 mg celecoxib, and morphine or tramadol to relieve pain. Every patient took the same postoperative rehabilitation plan. On the 7th day after the operation, they used crutches to carry partial weight and performed a series of training. Also every patient took the identical preoperative antibiotic regimen.

### 2.4 Outcome measures

Demographic characteristics collected effective information including gender, date of operation and other relevant information. The preoperative data was utilized to tell the history of past illness and operation status, body mass index, and chronic conditions. Postoperative outcome measures included operation time, total blood loss amount, validated mobility score, surgery-related syndrome, and tip-apex length. The postoperative X-ray pictures were taken on the second day after the operation, and the tip-to-tip distance was measured by an independent observer who was not involved in the operation of the designated patient as described above. The apex distance is defined as the total amount of the length from the tip of the lag screw to the top of the femoral head in anteroposterior and lateral views. The verified mobility score measures mobility in the range of 0 (no mobility) to 9 (full mobility).

### 2.5 Follow-up protocol

The patients were then re-examined at three time period: 3 weeks, 3 months, and 1 year. At the first period, remove skin sutures and evaluate wounds or other complications. At 3 months and 1 year, X-rays will be utilized to evaluate the healing of the fracture, the state of the implant, the progress of functional recovery. At the end of each postoperative period, staff will contact patients by phone and ask them to send a new hip X-ray and fill out a functional questionnaire.

### 2.6 Statistical analysis

All the relevant information and data were tabulated in Excel worksheets and analyzed using SPSS statistical software package. The independent sample t test was used to compare with the normally distributed continuous variables, while the Mann–Whitney *U* test was used to compare non-normally distributed data. Where appropriate, the Chi-square test or Fisher's exact test was utilized to compare categorical variables. The Kaplan–Meier survival analysis method was used to analyze the survival rate. Using the log-rank test to determine the independent impact of the type of equipment used on survival. The result of *P* < .05 was considered to be statistically significant.

## 3 Discussion

Our purpose for this study was to determine the most appropriate implant for a patient with a unstable intertrochanteric fractures. This study has several limitations, including its retrospective design. Given that hospital records are error prone, we attempted to contact each patient by phone to verify the hospital record data and achieve longer follow-up times. Although this method was fruitful in obtaining information on most of our patients, it was limited by the patients’ ability to recall events and their current cognitive status. Despite these limitations, we successfully contacted most of the patients. Finally, this study was limited in its ability to comment on functional outcomes.

## Author contributions

**Conceptualization:** Yu Bo.

**Data curation:** Yu Bo, Yue Qin.

**Formal analysis:** Yu Bo, Yue Qin.

**Funding acquisition:** Haibo Yang.

**Investigation:** Yu Bo, Yue Qin.

**Methodology:** Yu Bo, Yuan Zang.

**Project administration:** Haibo Yang.

**Resources:** Haibo Yang.

**Software:** Yue Qin.

**Supervision:** Haibo Yang.

**Validation:** Yue Qin, Yuan Zang.

**Writing – original draft:** Yu Bo.

**Writing – review & editing:** Yuan Zang, Haibo Yang.
